# Proprotein Convertase Subtilisin/Kexin Type 9 and Inflammation: An Updated Review

**DOI:** 10.3389/fcvm.2022.763516

**Published:** 2022-02-18

**Authors:** Na-Qiong Wu, Hui-Wei Shi, Jian-Jun Li

**Affiliations:** State Key Laboratory of Cardiovascular Diseases, Cardiometabolic Center, National Center for Cardiovascular Diseases, Fu Wai Hospital, Chinese Academy of Medical Sciences and Peking Union Medical College, Beijing, China

**Keywords:** PCSK9 (proprotein convertase subtilisin kexin type 9), inflammation, ASCVD, TLR4 (toll-like receptor 4), LOX-1

## Abstract

The function of Proprotein Convertase Subtilisin/Kexin Type 9 (PCSK9), a novel plasma protein, has mainly been involved in cholesterol metabolism in the liver, while, more interestingly, recent data have shown that PCSK9 also took part in the modulation of inflammation, which appeared to be another explanation for the reduction of cardiovascular risk by PCSK9 inhibition besides its significant effect on lowering lower-density lipoprotein cholesterol (LDL-C) concentration. Overall, a series of previous studies suggested an association of PCSK9 with inflammation. Firstly, PCSK9 is able to induce the secretion of proinflammatory cytokines in macrophages and in other various tissues and elevated serum PCSK9 levels could be observed in pro-inflammatory conditions, such as sepsis, acute coronary syndrome (ACS). Secondly, detailed signaling pathway studies indicated that PCSK9 positively regulated toll-like receptor 4 expression and inflammatory cytokines expression followed by nuclear factor-kappa B (NF-kB) activation, together with apoptosis and autophagy progression. Besides, PCSK9 enhanced and interacted with scavenger receptors (SRs) of inflammatory mediators like lectin-like oxidized-LDL receptor-1 (LOX-1) to promote inflammatory response. Additionally, several studies also suggested that the role of PCSK9 in atherogenesis was intertwined with inflammation and the interacting effect shown between PCSK9 and LOX-1 was involved in the inflammatory response of atherosclerosis. Finally, emerging clinical trials indicated that PCSK9 inhibitors could reduce more events in patients with ACS accompanied by increased inflammatory status, which might be involved in its attenuating impact on arterial plaque. Hence, further understanding of the relationship between PCSK9 and inflammation would be necessary to help prevent and manage the atherosclerotic cardiovascular disease (ASCVD) clinically. This review article will update the recent advances in the link of PCSK9 with inflammation.

## Introduction

Atherosclerotic cardiovascular disease (ASCVD) is a primary cause of morbidity and mortality around the world, which is definitely associated with multiple risk factors. Among them, the inflammation is the principal mechanism for the development of ASCVD except for lipid disorder. A large number of studies have demonstrated that chronic inflammatory response induced by substantial inherent or acquired risk factors exerts a significant effect on the initiation and development of atherosclerosis and the resulting plaque rupture and erosion, then it contributes to systemic repercussions of atherosclerosis-related cardiovascular diseases (CVD) (1). Besides, the interaction of risk factors such as lipid and inflammation has also been considered to play an important role in ASCVD. Interestingly, emerging data have shown that proprotein convertase subtilisin/kexin type 9 (PCSK9), an key protein of lipid metabolism, is involved in the production of both inflammatory cytokines and atherosclerosis plaque (2–7).

PCSK9 is primarily biosynthesized in the hepatocytes, then reaches the basolateral surface of the hepatocyte and binds low density lipoprotein receptor (LDLR) in an autocrine effect. Subsequently, the complex composed of LDL-C, LDLR, and PCSK9 is internalized into hepatocytes and undergoes endocytosis and lysosomal degradation, thereby reducing LDLR on the cell membrane and raising LDL-C levels (8). In addition of liver, PCSK9 is also expressed in many other tissues including small intestine, lung, kidney, pancreas and brain. Emerging studies have also found that PCSK9 is highly expressed in vascular endothelial cells (EC), smooth muscle cells (SMC) and macrophages (9), subsequently exerting local effects on vascular homeostasis and atherosclerotic plaques (10). Additionally, the detection of PCSK9 provides a new target for the management of hypercholesterolemia and the reduction of cardiovascular risk. Thus, the understandings of the physiology of PCSK9 have helped to broaden our knowledge in PCSK9 and PCSK9 inhibitors. Basically, although the mediation of up-regulation of LDLR accounts for the main effect of PCSK9 inhibitors, there is growing evidence supporting that PCSK9 may have a pleiotropy. One of these effects might be associated with inflammatory modulation in the development of ASCVD independent of LDLR regulation.

In this review, we generalized the biological characteristics of PCSK9 and mainly focused on updated evidence of the relation of PCSK9 to inflammation, in order that we could stress the clinical significance of the interaction between PCSK9 and inflammation.

## PCSK9 and Inflammation: Observational Cohort Evidence

Several previous studies examined the relation of PCSK9 to inflammation using cross-sectional observations. These studies mainly evaluated the correlation between plasma PCSK9 concentrations and a number of key inflammatory markers, including white blood cells (WBCs), fibrinogen, and high-sensitivity C-reactive protein (hs-CRP) (Table 1). For example, a small sample size study in Chinese patients with angiography-proven coronary artery disease (CAD) has shown that the increase of plasma PCSK9 level was associated with the elevation in white blood cell counts (WBCC), fibrinogen and high-sensitivity C-reactive protein (hs-CRP) (11). As is well-known, the WBCC is a traditional marker of inflammation and either inflammation or PCSK9 was considerably connected with atherosclerosis in populations with different levels of baseline risks. Interestingly, in a single-center study of stable CAD patients naïve to lipid-lowering therapy (6), both univariate and multivariate regression analyses showed that plasma PCSK9 levels were positively associated with WBCC subgroup, lymphocyte count and neutrophils count, while the molecular mechanism by which WBCC was associated with plasma PCSK9 levels was still unclear. Meanwhile, a cross-sectional study in stable CAD patients has revealed that circulating PCSK9 levels were positively correlated with fibrinogen levels but unrelated to some potential confounders such as lipid spectrometry and hs-CRP (2). Otherwise, a recent study indicated that PCSK9 was not induced in artificial human inflammation and was not correlated with inflammatory response in ten healthy volunteers stimulated by endotoxin (lipopolysaccharide, LPS) (12). It seemed that the study did not support the notion that PCSK9 could trigger an inflammatory response in human study.

**Table 1:** Correlation of plasma PCSK9 levels with inflammatory markers in patients with ASCVD.

**References**	**Features of clinical study**	**Inflammatory markers**	**Univariable analysis (*r*, *P*-value)**	**Multivariable analysis (β, *P*-value)**
Li et al. (6)	Single-center, cross-sectional study (251 stable CAD patients)	WBCC	0.167, *P* = 0.008	0.186, *P < * 0.01
Zhang et al. (2)	Cross-sectional study (219 stable CAD patients)	Fibrinogen Hs-CRP	0.211, *P* = 0.002 0.153, *P* = 0.023	0.168, – 0.011, –
Gencer et al. (7)	Multi-center prospective cohort study (2,168 ACS patients)	Hs-CRP	0.077, *P* = 0.006	–, – –, –
Li et al. (11)	Prospective study (552 CAD patients)	WBCC Fibrinogen	0.077, *P* = 0.004 0.181, *P* < 0.001	–, – –, –
Heinzl et al. (12)	Prospective, single-blinded, placebo-controlled cross-over study (10 healthy nonsmoking male receiving LPS or placebo)	IL-6 CRP PCSK9 PCSK9 and IL-6	*P* = 0.018 (RM-ANOVA) *P* < 0.001(–) *P* = 0.44(–) *P* = 0.358	–, – –, – –, – –, –

CRP, a kind of acute phase mediator, is considered to be a systemic inflammatory biomarker of sensitivity but of no specificity. The elevation in plasma hs-CRP concentration has also been considered to be the risk factor of atherosclerosis (17). Observational studies have revealed that plasma CRP levels were a powerful predictor for cardiovascular (CV) risk and logarithmically correlated with CAD risk (18). Recently, Pradhan et al. assessed the residual risk of inflammation in 9,738 patients who had received both statins and bococizumab in the Studies of PCSK9 Inhibition and the Reduction of Vascular Events (SPIRE)-1 and SPIRE-2 cardiovascular outcomes trial. They evaluated residual risk according to on-treatment levels of hsCRP(hsCRP_OT_) recorded 14 weeks after drug initiation. The data indicated that increased hsCRP_OT_ remained as an important predictor of cardiovascular risk in CAD patients receiving statins and PCSK9 inhibitors (19). This study suggested that although the maximum reduction in LDL-C was achieved, regulating inflammation provided additional opportunities to reduce cardiovascular risk, which was named as residual inflammatory risk.

Furthermore, in patients with stable CAD, PCSK9 levels were significantly positively correlated with hs-CRP levels (2). Recently, in a large prospective multi-center study conducted in patients with acute coronary syndrome (ACS), patients with higher levels of circulating PCSK9 suffered a higher degree of acute phase inflammation assessed by hs-CRP levels (7). Similarly, a prospective case-control study in CAD patients naïve to lipid-lowering therapy showed a significantly positive correlation between PCSK9 levels and the incidence and severity of CAD (11), and the effect of PCSK9 on CAD is primarily mediated by the increased atherogenic lipids and inflammatory markers. Hence, these studies have shown that PCSK9 is associated with the occurrence of inflammation in the occurrence and development of CAD, suggesting that inhibition of PCSK9 may have a therapeutic effect on atherosclerotic inflammation and CAD. However, although there was no doubt that increased plasma PCSK9 concentrations were in association with elevated inflammatory biomarkers, whether PCSK9 is causal trigger for inflammation might need to be further confirmed.

## PCSK9 and Inflammation: Basic Investigations in Atherosclerosis

Although the relation of PCSK9 to the formation of atherosclerotic plaque is unclear, several basic studies showed that PCSK9 might be involved in the development of atherosclerotic plaque through inflammation-mediated process.

It is well-known that ASCVD is an inflammatory process. A previous study using multilocal positron emission tomography- magnetic resonance imaging suggested an arterial inflammation in patients with sub-clinical atherosclerosis, so it convincingly revealed an inflammatory state in the early stages of atherosclerosis (20). Chronic inflammation, along with other factors such as high blood pressure, diabetes and smoking, has become the ultimate critical pathway leading to the development and progression of ASCVD. Data also showed that the activation of endothelium could lead to the secretion of surface molecules which were subsequently adsorbed into inflammatory cells, following monocytes' and macrophages' migration across the endothelium and accumulation beneath the intima. Subsequently, these cells release cytokines and produce a pro-inflammatory environment during activation. With time going by, Lectin-like OXLDL Receptor-1 (LOX-1) combines with circulating oxidized LDL (ox-LDL) on vascular smooth muscle cells (VSMCs) and monocytes/macrophages and enters the vascular stroma, then it results in the formation of foam cells (21, 22). In addition, ischemic myocardium is characterized by the release of pro-inflammatory cytokines into the blood, which causes a violent inflammatory response. In patients with myocardial ischemia, especially in the acute phase, pro-inflammatory biological factors such as hsCRP, tumor necrosis factor-α (TNF-α), interleukin-6 (IL-6), and interleukin-1β (IL-1β) were significantly increased (23).

**Figure 1:**
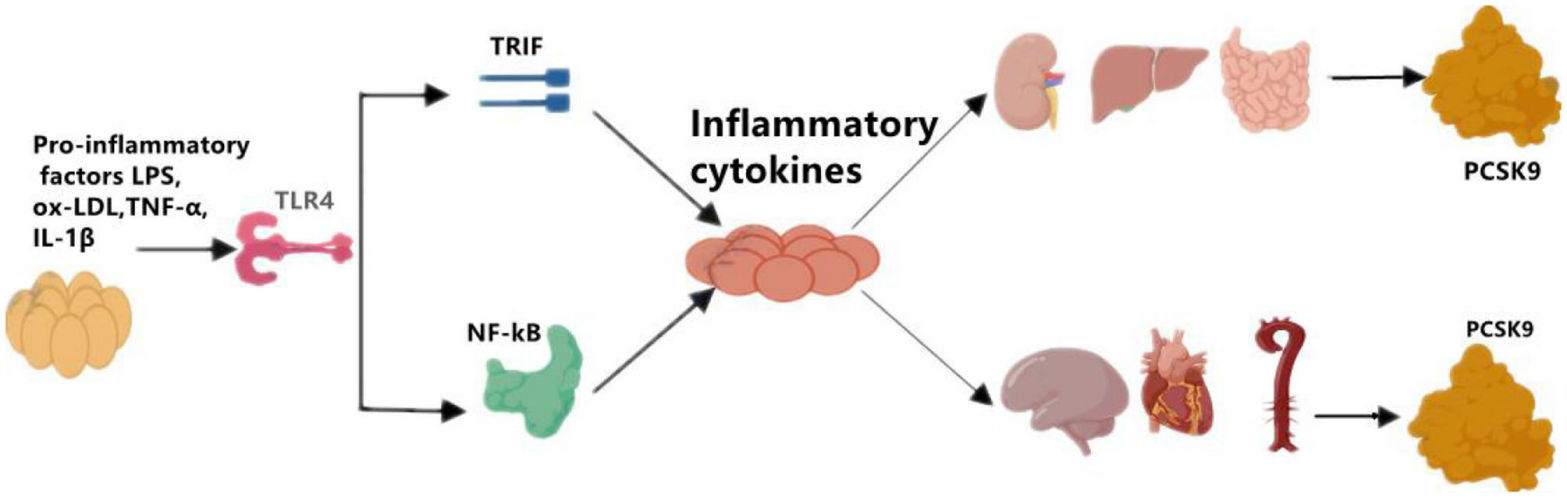
The regulation of on PCSK9 secretion. Pro-inflammatory factors, such as LPS, ox-LDL, TNF-α, and IL-1β induces the expression of PCSK9 in main sources for PCSK9 such as liver, kidney, small intestine, and in brain, heart, artery as well. LPS, lipopolysaccharide; oxLDL, oxidized low-density lipoprotein; TNF-α, tumor necrosis factor alpha; IL-1β, interleukin 1β; TLR4, toll-like receptor 4; NF-kB, nuclear factor kappa-light-chain-enhancer of activated B cells; PCSK9, proprotein convertase subtilisin/kexin Type 9.

Although the primary sources of PCSK9 are hepatocytes, other cells in extrahepatic tissues such as the brain, heart, kidney, small intestine, and blood vessels can also produce PCSK9 that is secreted into the circulation (Figure 1). Epicardial adipose tissue (EAT) could also be a source of PCSK9 and EAT inflammation was correlated with local PCSK9 expression (24). The expression of PCSK9 in vascular endothelial cells (EC) and VSMCs is mainly regulated by proinflammatory stimulation such as ox-LDL, TNF-α, IL-1β, and LPS (25). Studies have shown that plasma levels of PCSK9 were associated with circulating LDL-C as well as some other risk factors for coronary diseases, and that high levels of PCSK9 have been found in patients suffering systemic inflammatory response syndrome (SIRS) and sepsis (26). Moreover, The European Collaborative Project on Inflammation and Vascular Wall Remodeling in Atherosclerosis-Intravascular Ultrasound (ATHEROREMO-IVUS) study showed that serum PCSK9 levels were in relation to the absolute volume of inflammatory plaque and necrotic core tissue (27). In previous studies, Almontashiri et al. observed that elevated serum PCSK9 levels were presented in patients with acute myocardial infarction and myocardial ischemia, especially in those newly diagnosed (28). In their studies, a significant association of serum PCSK9 levels with pro-inflammatory cytokines IL-6, IL-1β, TNF, MCSF (macrophage colony-stimulating factor) and hs-CRP was found (28). Experimental data showed that in the vascular injury model of PCSK9^−/−^ mouse, the loss of PCSK9 was linked to the reduction of neointima formation in atherosclerotic plaques (5). Other experimental studies about the impact of PCSK9 on atherosclerotic plaques formation were shown in Table 2.

**Table 2:** Experimental studies about the impact of PCSK9 on atherosclerotic plaques formation.

**References**	**Model**	**Treatment**	**Impact on systemic inflammation**	**Impact on vascular inflammation**
Giunzioni et al. (10)	ApoE^−/−^ recipient mice	hPCSK9	Raised Infiltration of Ly6C(hi) inflammatory monocytes (+32%)	–
Tavori et al. (13)	LDLR^−/−^ or apoE^−/−^ mice overexpressing human PCSK9 (Hpcsk9) model	hPCSK9	–	1. Increased atherosclerotic lesion size and composition independent of lipids and lipoprotein changes 2. Increased accumulation of PCSK9 in the arterial wall
Ferri et al. (5)	PCSK9^−/−^ mice model	Deletion of PCSK9 gene	–	1. Attenuated neointimal plaque formation 2. Higher SMCs accumulation
Sun et al. (14)	Atherosclerosis-prone mouse model	Deletion of PCSK9 gene	–	1. Reduced atherogenesis 2. Decreased expression of adhesion molecules, ICAM-1, MCP-1, and MCP-3 by ECs
Kühnast et al. (15)	APOE*3Leiden.CETP mice	Alirocumab	Decreased pro-inflammatory Ly6Chi monocytes	1. Attenuated vascular inflammation and necrotic core formation 2. Improved plaque stability 3. Reduced expression of ICAM-1 in ECs
Landlinger et al. (16)	APOE*3Leiden.CETP mice	Anti-PCSK9 vaccine	Decreased serum levels of MCSF-1 and VEGF-A	1. Reduced plaque number and size 2. Decreased plasma level of ICAM-1 in ECs

Notably, PCSK9 can regulate LDLR expression locally in neighboring cells including arterial monocytes/macrophages (29). PCSK9 overexpression could result in increased size of atherosclerotic plaques, the phenomenon of which had not been observed in LDLR^−/−^ mice, suggesting that the role of PCSK9 in atherosclerotic development was related to LDLR (10). In an investigation elucidating the direct pro-atherogenic role of PCSK9 in atherosclerosis, firstly, WT mice expressing null (KO) level of PCSK9 accumulated 4-fold less aortic cholesteryl esters (CE) than WT mice, whereas mice expressing high (Tg) levels of PCSK9 exhibited high CE and severe aortic lesions. In addition, apoE-deficient mice that expressed null (KO/e) levels of PCSK9 showed a 39% reduction in aortic CE accumulation compared to those expressing normal (WT/e) levels of PCSK9, while Tg/e mice showed a 137% increase. Finally, LDLR-deficient mice expressing null (KO/L) and high (Tg/L) levels of PCSK9 exhibited similar levels of plasma cholesterol and CE accumulation to WT/L, suggesting that PCSK9 modulated atherosclerosis mainly via the LDLR (30).

Obviously, these studies have confirmed that PCSK9 enhances the infiltration of inflammatory monocytes into the vessel wall by virtue of the interaction of PCSK9-LDLR in plaques, which thus directly promotes the formation of inflammatory atherosclerotic plaques. In addition of LDLR, other members of the LDLR superfamily such as LRP5 may also be a target of PCSK9. A study focusing on primary cultures of inflammatory cells including monocytes and macrophages found that LRP5 and LDLR acted through different mechanisms. Since for one, no variation in LDLR expression levels existed in control cells but did in LRP5-silenced cells and LRP5 was not regulated by lipoprotein receptor modulator SREBP-2, for another, in PCSK9-silenced macrophages, LDLR expression increased significantly after agLDL loading but LRP5 levels didn't alter. The study also observed that PCSK9 binds LRP5 at the perinuclear area of human macrophages and the two form a complex located in the cytoplasm of macrophages, and this interaction was involved in lipid uptaking in macrophages. In addition, LRP5-silenced macrophages showed a reduced release of PCSK9, demonstrating that LRP5 participates in the release of PCSK9. Further, in macrophages silenced for both LRP5 and PCSK9, reduction in CE accumulation was observed. Moreover, in PCSK9 silenced-macrophages, decreased TLR4 protein levels and rescued increase in TNFα and IL-1β showed, revealing a role of PCSK9 in macrophage inflammation associated to the TLR4/NFκB pathway (31).

Meanwhile, a clinical study indicated that elevated serum levels of PCSK9 were associated with new plaque formation even after adjusting LDL-C levels and other traditional risk factors (3). Therefore, the pro-atherosclerotic effect of PCSK9 was not only related to the disturbance of lipid metabolism but also intertwined with PCSK9-stimulated plaque inflammation, which was further supported in ApoE^−/−^ or LDLR^−/−^ transgenic mice that overexpressed human PCSK9 (10). This study might provide additional evidence of the local effect of PCSK9 on inflammatory plaques, indicating the direct role of PCSK9 in atherosclerotic plaques. That is, PCSK9 expressed from bone-marrow derived macrophages could directly and locally accentuate vascular inflammation by changing the composition of lesion, but not by changing the lesion size and serum cholesterol level (10). Interestingly, *in vitro* studies have also confirmed the association of PCSK9 with monocyte-mediated plaque inflammation, suggesting that local PCSK9 production by VSMCs could inhibit C-C chemokine receptor type 2(CCR2)-dependent chemotaxis of monocytes in plaques, thereby enhancing their sustaining expression in the atherosclerotic environment (32). In addition, pro-inflammatory leucocytes played a critical role in atherosclerotic development and at the same time regulated the composition of atheroma lesion while no significant changes in cholesterol levels and lesion size were observed (33). Thus, these findings demonstrated that through altering plaque composition and accelerating inflammatory monocytes infiltration and differentiation in plaques, PCSK9 could directly promote atherosclerotic inflammation independently of cholesterol regulation, which indirectly supported the notion that PCSK9 inhibitors can improve clinical outcomes through not only lipid dependent but also lipid independent pathways.

## PCSK9 and Inflammation: Potential Signaling Pathways

Although PCSK9 has been considered as a trigger for the expression of pro-inflammatory cytokines, the detailed mechanism it involves remains to be summarized. There appeared two associated signaling pathways involved in the positive regulation of PCSK9 to inflammatory cytokines expression and atherosclerotic lesions formation.

Firstly, the Toll-like receptor 4(TLR4)/nuclear factor-kappa B (NF-*k*B) signaling pathway has been found to be the main pathway that mediates PCSK9-induced expression of pro-inflammatory cytokine (34), and plays an indispensable role in the initiation and development of atherosclerotic lesions by inducing vascular inflammation (35). TLR4 stimulates the activation of NF-kB transcription factor, which is obligated to producing many pro-inflammatory genes, including TNF-α, IL-6, interleukin(IL)-1, and macrophage chemotactic protein 1 (MCP-1) (36). Primarily functioning through regulating inflammatory response, NF-*k*B is a Redox sensitive transcription factor which can be activated by a variety of stimuli, including oxidized LDL (ox-LDL), reactive oxygen species (ROS), Toll-like receptor (TLR), cytokines, and bacterial products such as LPS. *In vitro* studies in RAW264.7 macrophages stimulated by ox-LDL also identified the involvement of the TLR4/NF-*k*B signaling pathway in PCSK9-mediated inflammation. According to their study, up-regulation and down-regulation of PCSK9, respectively, increased and decreased ox-LDL-induced expression of pro-inflammatory cytokines including TNF-α, IL-1β and MCP-1. This outcome is related to the up-regulation of TLR4 expression triggered by ox-LDL, followed by nuclear translocation of NF-*k*B (34). Basically, PCSK9 is most likely to increase the expression of pro-inflammatory cytokines through combining with the C-terminal domain of TLR4, resulting in increased TLR4 expression as well as activated TLR4/NF-*k*B signaling pathway (34).

**Figure 2:**
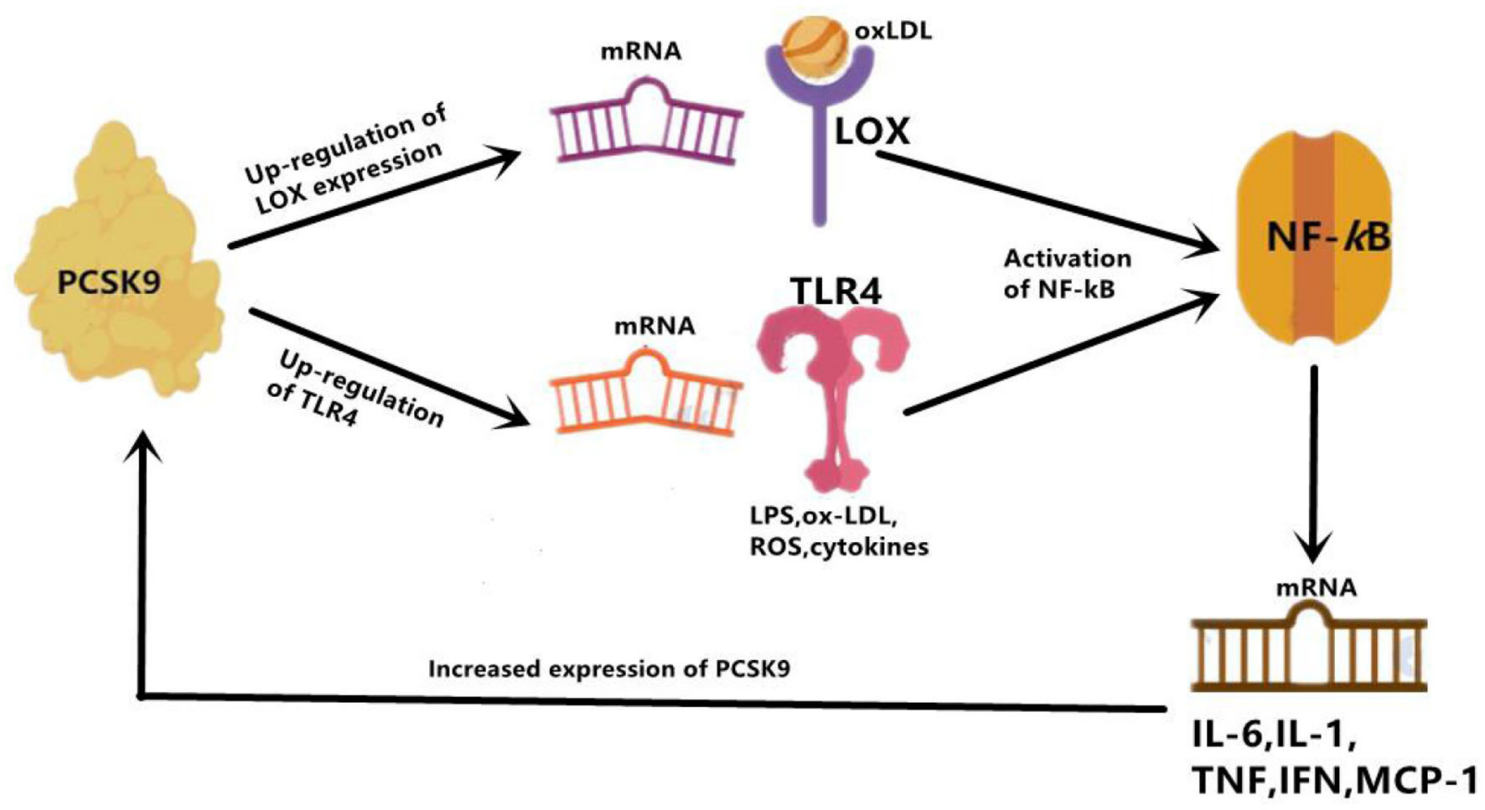
PCSK9 mediate inflammation through enhancing expression of TLR4 and LOX. LOX can mainly increase uptake of ox-LDL, and TLR4 increase uptake of LPS. Then activation of NF-kB follows. Overall, activated NF-kB up-regulates expression of inflammatory cytokines, such as IL-6, IL-1, TNF-α, IFN- γ, and MCP-1. IL-1, interleukin 1; IL-6, interleukin 6; IFN-γ, interferon gamma; MCP-1, monocyte chemoattractant protein 1.

The effects of PCSK9 on TLR4/NF-kB regulated inflammation has also been verified in a study with LPS-induced sepsis model, in which LPS could induce inflammatory response by virtue of increased PCSK9 (37). The interaction of LPS and PCSK9 may be explained by previous facts. LPS could induce TLR4 and trigger NF-kB signaling pathway, at the same time it up-regulates PCSK9, then leads to systemic inflammation (38), which might indicate that the pro-inflammatory effect of PCSK9 may be intermediated, at least in proportion, by targeting the activation of the TLR4/NF-kB pathway (38). Another previous study also showed that PCSK9 over-expression could increase plasma IL-6 concentration, while knockout of PCSK9 could decrease plasma IL-6 levels and attenuate organ inflammatory response in the mouse septicemia model (39). Similar results were also reported by another study on PCSK9 knockout mice. In their study, data showed an attenuated impact on LPS-induced inflammation and decreased plasma levels of pro-inflammatory cytokines such as tumor necrosis factor-α (TNF-α), IL-6, MCP-1, and macrophage inflammatory protein 2(MIP-2) (40). Results from a human study further supported that patients with septic shock who carried the PCSK9 loss-of-function (LOF) allele had lower serum levels of pro-inflammatory cytokines compared with patients with the gain-of-function (GOF) allele (40). These findings indicated an association of PCSK9 with inflammation in LPS-induced sepsis model, suggesting that PCSK9 would play a role of pro-inflammatory mediator. Moreover, in the study we have discussed at the beginning of this paragraph, down-regulation of PCSK9 inhibitor Farnesoid X receptor and peroxisome proliferation-activated receptor alpha (PPARα) transcription factor could increase PCSK9 expression, resulting in a decrease in liver LDLR expression and an increase in plasma LDL-C (37) (Figure 2).

Next, the activation of the PCSK9-LOX-1 axis has also been demonstrated to participate in PCSK9-mediated inflammatory response. During the formation of atherosclerotic plaque, circulating oxidized LDL (ox-LDL) is bound to scavenger receptors (SRs) of inflammatory mediators like LOX-1 locating on the surface of endothelial cells (ECs) (22). As a well-recognized mediator of inflammation and atherosclerosis (21, 41, 42), LOX-1 is the principal receptor for ox-LDL on ECs and VSMCs, and is expressed when macrophages, SMCs, and fibroblasts are exposed to ox-LDL, angiotensin II, or proinflammatory cytokines. Studies have shown that there is a positive feedback between PCSK9 and LOX-1 in VSMCs. The activation of LOX-1 stimulates the expression of PCSK9 (25), and in contrast, PCSK9 promotes the expression of LOX-1 and the uptake of ox-LDL, which triggers a pro-inflammatory state. Additionally, pathological studies did suggest that LOX-1 and PCSK9 were co-expressed in atherosclerotic plaques, indicting that PCSK9 and LOX-1 may interact with each other in the inflammatory microenvironment (25). Notably, LOX-1 is highly expressed in growing plaques and ruptured plaques (41) and also in ischemic heart, leading to inflammation and cardiomyocyte apoptosis (43). And also, acting as a primary NF-kB activator, ox-LDL induces inflammatory response in EC and macrophages and enhances the expression of PCSK9 (44). In contrast, down-regulation of PCSK9 could reduce ox-LDL-induced inflammatory response accompanied by reduction in pro-inflammatory cytokines including IL-1α, IL-6, and TNF-α (44). Laboratory studies on LOX-1 gene deletion and LDLR knockout mice have shown a significant reduction in atherosclerosis progression (45), which might be in relation to a critical reduction in the accumulation of inflammatory cells in the vessel wall. On the contrary, LOX-1 overexpression in ECs could accentuate plaque formation and atherosclerotic progression (46).

The interplay between PCSK9 and LOX-1 may also be explained by the regulation of mitochondrial ROS (mtROS) and NF-kB (47). Interestingly, VSMC-originated PCSK9 was shown to induce the damage of mitochondrial DNA, the fragments of which could promote mtROS-mediated expression of PCSK9/LOX-1 (48). Both *in vitro* and *in vivo* studies showed that changes in ROS production and fluid shear force would activate the PCSK9-LOX-1 axis (47). Mechanically, the regulation of fluid shear stress on PCSK9 expression was mediated by Nicotinamide Adenine Dinucleotide Phosphate (NADPH) oxidase-dependent ROS production in VSMCs and ECs of human and mouse aorta (47). Meanwhile, evidence showed that the two-way crossover linking ROS production and PCSK9 expression may be mediated by the NADPH oxidase system in aortic tissue under inflammatory state, thereby regulating the deposition of LDL and ox-LDL in atherosclerotic areas (49). In general, under low shear stress conditions, such as in the inflammatory state, PCSK9 could enhance inflammatory response in atherosclerotic lesion through activation of the ROS/NF-kB/LOX-1/oxLDL axis in VSMCs.

In summary, PCSK9 up-regulates the expression of TLR4 and LOX-1, both of which further activates NF-kB and induces the expression of inflammatory cytokines. Thus, TLR4/NF-kB axis and PCSK9/LOX-1/NF-kB axis are the two mainly involved signaling pathways mediating the PCSK9-induced pro-inflammatory conditions.

## PCSK9 Inhibitors and Inflammation: Clinical and Experimental Observations

Both experimental results and clinical trials indicated the repressive role of PCSK9 inhibition on vascular inflammation and subsequent development of atherosclerosis.

**Figure 3:**
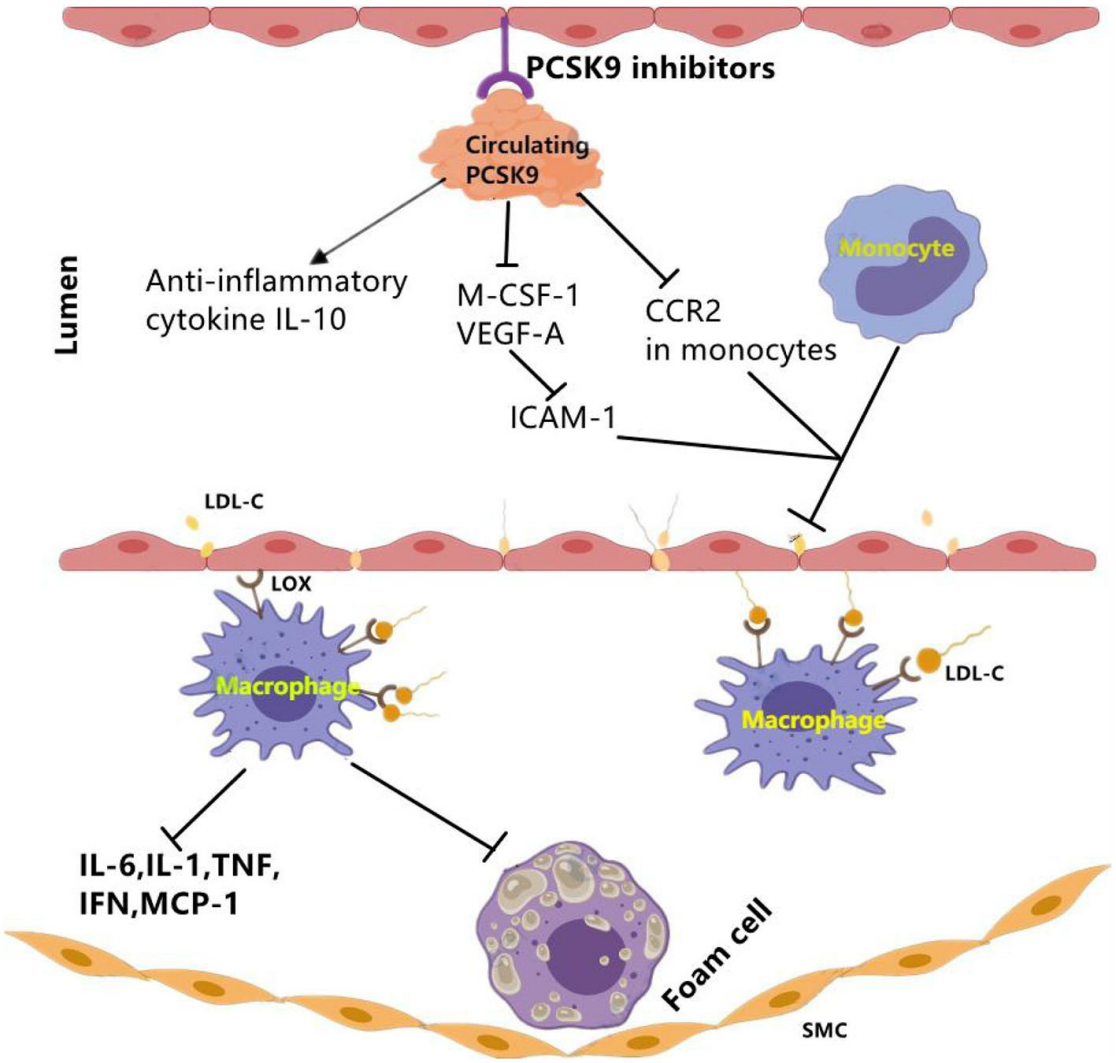
The effect of PCSK9 inhibition on vascular inflammation. PCSK9 inhibition could decrease the expression of main markers of vascular inflammation including M-CSF-1 and VEGF-A which leads to reduced ICAM-1 expression in endothelial cells and reduced infiltration of monocytes into the subendothelial layer. Moveover, PCSK9 inhibition reduces CCR2 expression and the inhibition of monocyte migration. Besides pro-inflammatory mediators, PCSK9 inhibitors may function through elevating anti-inflammatory cytokines such as IL-10. CCR2, C–C chemokine receptor Type 2; ICAM-1, intercellular adhesion molecule 1; LDL-C, low-density lipoprotein cholesterol; LOX-1, lectin-like oxidized LDL receptor-1; MCSF-1, major histocompatibility complex; oxLDL, oxidized low-density lipoprotein; SMCs, smooth muscle cells; VEGF-A, vascular endothelial growth factor A. The signal “→”indicates “promotes,” “⊣”indicates “inhibits”.

PCSK9 antagonists could be achieved by active vaccination which binds to PCSK9 and inhibits its interaction with LDLR. In APOE^*^3 Leiden.cholesteryl ester transfer protein (CETP) mice study (16), data showed that PCSK9 AT04A vaccine could exert immunosuppression to circulating PCSK9, and reduce the plasma cholesterol levels by 53% (16), and decrease the concentrations of macrophage colony stimulating factor 1(M-CSF-1), vascular endothelial growth factor A(VEGF-A). Then, reduced plasma levels of M-CSF-1 and VEGF-A led to decreased expression of intracellular adhesion molecule-1 (ICAM-1) in endothelial cells, thereby reducing recruitment and adhesion of monocytes to the vascular endothelium (16) (Figure 3). Moreover, a decrease in the number and size of atherosclerotic plaques was also observed in AT04A-inoculated mice (16). Besides, in a prospective, observational, multicenter trial involving 21 consecutive patients with stable CVD, researchers evaluated arterial inflammation using 18F-fluoro-2-deoxy-D-glucose.

(FDG) positron emission tomography/computed tomography (PET/CT). They found that long-term administration of PCSK9 inhibitor significantly improved arterial inflammation, and that in index vessel, target-to-background ratio (TBR) detected by PET/CT significantly decreased by 0.92 (95% CI: 0.56, 1.28, *P* < 0.001) (50).

In addition of clinical data, there was also experimental data from an *in vivo* study using ApoE ^*^3Leiden.CETP mice. This study found that alirocumab reduced endothelial expression of ICAM-1 and Ly6C^hi^ monocytes (Ly6C^hi^ monocytes are the precursors of proinflammatory M1 macrophages, and they would progress into pro-inflammatory M1 macrophages) (15). The results also showed the decrease of other markers for vascular inflammation including T cell abundance in the aortic root region, necrotic content in macrophages, cholesterol division in arterial plaques as well (15). Briefly, alirocumab can significantly improve morphology and stability of lesion in mouse models of atherosclerosis (15).

In addition of intracellular accumulation of lipids in monocytes, a clinical study carried out in patients with Familial hypercholesterolemia (FH) also observed that PCSK9 inhibition decreased chemokine receptor Type-2(CCR2) expression which correlated with diminished trans-endothelial migratory capability of monocytes (51). Meanwhile, reduction in TNF-α levels and elevation in anti-inflammatory cytokine IL-10 were also revealed (51). Further evidence from clinical trials showed an anti-inflammatory role of evolocumab, alirocumab, and bococizumab in patients with stable CAD patients (19, 52) and patients living with HIVs (PLWH) and People With Dyslipidemia (53) (Table 3).

**Table 3:** Percentage changes of hsCRP and LDL-C after PCSK9 inhibition agents treatments.

**clinical study**	**PCSK9 inhibition agents**	**Hs-CRP (mg/L)**			**LDL-C (mg/dL)**		
		**Baseline**	**Post**	**Percentage change**	**Baseline**	**Post**	**Percentage change**
FOURIER (52)	Evolocumab	1.7	1.4	0% vs. placebo	92.0	30.0	−59% vs. placebo
ODYSSEY COMBOII (54)	Alirocumab	3.58	3.51	−2% vs. baseline	108.0	53.3	−49.5% vs. baseline
ODYSSEY OUTCOMES (55)	Alirocumab	1.6	NA	NA	87.0	53.0	−54.7% vs. placebo
Single-Center study (53)	Evolocumab	1.81	2.46	+35.9% vs. baseline	118.0	64.0	−45.7% vs. baseline
*Post-hoc* analysis of the SPIRE trials (19)	Bococizumab	1.88	1.84	+6.6% vs. placebo	96.5	34.7	−60.5% vs. placebo

All these studies indicated that systemic and vascular inflammation and the development of atherosclerosis were ameliorated by PCSK9 inhibitors. However, PCSK9 inhibitors failed to affect the level of hs-CRP in some studies.

Results of *post-hoc* analysis of the SPIRE trials of bococizumab (19) showed that plasma levels of hs-CRP measured 14 weeks after drug initiation did not decrease as expected (+6.6%), while circulating LDL-C markedly reduced by 60.5%. In a placebo-controlled, double-blind study, 14 weeks of alirocumab treatment resulted in a robust reduction in arterial wall inflammation and marked LDL-C–lowering in high CV risk patients, but no changes were observed in the plasma inflammatory markers (56). Besides, in a randomized, double-blind, and dose-ranging phase 2 Study conducted in patients with CHD, PCSK9 inhibitor RG7652 treatment led to a significant dose-dependent decrease in LDL-C level, but failed to bring in significant reductions in circulating systemic markers such as hs CRP, IL-6, and TNF-α (57). More convincingly, a meta-analysis of randomized controlled trials assessing the impact of PCSK9 inhibitors also concluded that there was no significant impact on circulating level of hs-CRP (13).

## PCSK9 and Inflammation: Conclusion

As is well-known, ASCVD is a leading cause of mortality in the world and lipid metabolic disorder and inflammation are two principal triggers for the development of ASCVD. PCSK9 as an emerging novel target for LDL-C catabolism has widely been well-recognized since discovery via parallel molecular biology and clinical genetics studies in 2003. Following studies to characterize PCSK9 has shed new light on its multiple effects of cardiovascular system. One of them is the interaction between PCSK9 and inflammation.

The pro-inflammatory role of PCSK9 in atherosclerosis progression has been confirmed both by experimental evidence and clinical data. Animal models confirmed that PCSK9 gene expression could affect serum levels of systemic inflammatory cytokines such as IFN-c, TNF-a, IL-6, and MCP-1. Observations in recent years from clinical studies found that in patients with ACS and CAD, elevated plasma levels of PCSK9 were independently linked to major systemic inflammatory markers including WBCs, hs-CRP, and fibrinogen. At the same time, there still are abundant experimental and clinical data investigating the consequences of PCSK9 inhibition on systemic and vascular inflammation. Atherosclerosis models exhibited that PCSK9 inhibition restrains atherosclerotic progression and improves plaque morphology. Clinical data showed that alirocumab therapy exerted local anti-inflammatory effect through decreasing the expression of CCR2 and anti-inflammatory cytokines. These discoveries regarding the relation of PCSK9 to inflammation have refreshed our understandings of the PCSK9 and PCSK9 inhibitors, which may help to promote a new era of cardiovascular disease prevention.

As a consequence, further studies may be needed to be carried out to explore the direct anti-inflammatory effect of PCSK9 inhibitors irrespective of LDL-C reduction. Exploring the connections of PCSK9 inhibition, amelioration of inflammation, and CV risk reduction by virtue of investigations from future studies and *post-hoc* analyses of long-term clinical trials are also of necessity.

## Author Contributions

J-JL was the originator, supervisor of the project, and conducted elaborate polishment on the paper. N-QW and H-WS collected and analyzed relevant literature, then completed the writing of the first draft of the paper. All authors read and agree with the final manuscript.

## Funding

This study was partly supported by Capital Health Development Fund (201614035) and Chinese Academy of Medical Sciences Innovation Fund for Medical Sciences (2016-I2M-1-011) awarded to J-JL.

## Conflict of Interest

The authors declare that the research was conducted in the absence of any commercial or financial relationships that could be construed as a potential conflict of interest.

## Publisher's Note

All claims expressed in this article are solely those of the authors and do not necessarily represent those of their affiliated organizations, or those of the publisher, the editors and the reviewers. Any product that may be evaluated in this article, or claim that may be made by its manufacturer, is not guaranteed or endorsed by the publisher.
